# Role of transanal tube placement in preventing anastomotic leakage in rectal cancer surgery with sufficient perfusion confirmed by indocyanine green fluorescence imaging

**DOI:** 10.1007/s10151-025-03255-8

**Published:** 2025-12-24

**Authors:** Koichiro Okada, Gaku Ohira, Ryota Miura, Toru Tochigi, Tetsuro Maruyama, Atsushi Hirata, Michihiro Maruyama, Hisahiro Matsubara

**Affiliations:** Department of Frontier Surgery, Chiba University Graduate School of Medicine, 1-8-1 Inohana, Chuuou-Ku, Chiba, 260-8670 Japan

**Keywords:** Anastomotic leakage, Rectal cancer surgery, Transanal tube, Indocyanine green fluorescence angiography, Anastomotic perfusion

## Abstract

**Background:**

Anastomotic leakage (AL) remains a major complication after rectal cancer surgery. Although various techniques have been proposed to reduce its incidence, the effectiveness of transanal tube (TA) placement remains controversial. This study aimed to evaluate whether combining indocyanine green (ICG) fluorescence angiography with TA placement reduces the risk of AL after rectal cancer surgery.

**Methods:**

A retrospective analysis, including patients who underwent rectal resection with primary anastomosis for rectal cancer, was performed. In all cases, anastomotic perfusion was assessed intraoperatively using ICG fluorescence angiography. Patients were categorized into two groups on the basis of postoperative TA placement: TA group and non-TA group. The primary outcome was the incidence of AL. Multivariate logistic regression and subgroup analyses based on tumor location were performed.

**Results:**

The TA group demonstrated a significantly lower incidence of AL compared with the control group (5% versus 18%, *p* = 0.02). Multivariate analysis identified male sex as a risk factor and TA placement as a protective factor for AL. Subgroup analysis revealed that TA placement was particularly effective in patients with middle rectal cancer.

**Conclusions:**

The placement of a TA may offer additional benefit in reducing the risk of AL after rectal cancer surgery when adequate perfusion is confirmed using ICG fluorescence imaging, particularly in cases of middle rectal cancer.

## Introduction

Rectal cancer is one of the most prevalent malignant tumors worldwide and is associated with high morbidity and mortality rates [1]. Advances in surgical techniques, particularly the introduction of total mesorectal excision (TME) over conventional blunt dissection, have significantly improved both local control and long-term survival [2]. TME has become the standard approach for rectal cancer surgery, as it reduces local recurrence, preserves the pelvic autonomic nerve plexus, and improves oncological outcomes [3].

However, despite these advancements, anastomotic leakage (AL) remains one of the most concerning complications in rectal surgery, given its detrimental effects on postoperative recovery, quality of life, and overall survival [4]. Multiple factors have been implicated in AL development, including anastomotic tension, compromised vascular perfusion at the anastomotic site, and elevated intraluminal pressure [5].

To mitigate the risk of AL, several intraoperative strategies have been developed to ensure adequate perfusion of the anastomotic site. Among these, near-infrared fluorescence imaging using indocyanine green (ICG) allows real-time visualization of bowel perfusion during minimally invasive surgery and has been recognized as a reliable tool for assessing vascular integrity and guiding surgical decision-making [5].

Several studies have demonstrated that ICG-based perfusion assessment can reduce the incidence of AL by enabling surgeons to adjust the transection line on the basis of real-time blood flow evaluation [6–8]. Nevertheless, AL still occurs even after sufficient perfusion has been confirmed using ICG, suggesting that factors beyond vascular supply, such as intraluminal pressure or anastomotic tension, may contribute to its development.

Recently, transanal tube (TA) placement has been proposed as an adjunctive technique to mitigate the risk of AL, particularly by lowering intraluminal pressure at the anastomotic site and facilitating smoother passage of intestinal contents [9, 10].

Despite promising results from several observational studies, the clinical utility of TA remains controversial, and its role in patients with adequate perfusion, as confirmed by ICG imaging, has not been fully established. Importantly, few studies have specifically examined the effectiveness of TA drainage in patients with adequate anastomotic perfusion confirmed by ICG fluorescence imaging. This represents a critical gap in the current evidence.

Therefore, this study aimed to evaluate the potential of TA placement in reducing the risk of AL in patients with rectal cancer undergoing resection with adequate anastomotic perfusion confirmed by ICG fluorescence imaging.

## Methods

### Study design and patient selection

A retrospective analysis was performed using a prospectively maintained database of consecutive patients who underwent rectal cancer surgery at Chiba University Hospital between January 2014 and May 2024. Among these, patients who underwent low anterior resection with intraoperative assessment of anastomotic perfusion using ICG fluorescence imaging were included in the study. Patients who underwent coloanal anastomosis, total proctocolectomy, or construction of a diverting stoma, as well as those who underwent emergency surgery or surgery without anastomosis, were excluded from the analysis. A total of 211 patients met the inclusion criteria and were categorized into two groups: those who underwent TA placement (TA group, *n* = 56) and those who did not (non-TA group, *n* = 155). This study was approved by the local Institutional Ethics Review Board (approval number: HK202411-11), and informed consent was not required due to its retrospective nature.

### Data collection and variables

The following variables were extracted from the prospectively maintained institutional database:

- Patient demographics: age, sex, and American Society of Anesthesiologists physical status classification.
- Tumor-related factors: tumor location (distance from the anal verge), tumor size (maximum diameter), and pathological stage according to the tumor node metastasis (TNM) classification.
- Surgical details: surgical approach (open versus minimally invasive), whether lateral lymph node dissection was performed, left colic artery preservation, operative time, and estimated blood loss.
- Treatment-related factors: administration of neoadjuvant therapy (chemoradiotherapy or chemotherapy).

Postoperative outcomes included the incidence of AL, defined as a clinically evident dehiscence of the anastomosis, diagnosed through abdominal computed tomography scan or enterography, or the presence of fecal discharge through the pelvic drain within 90 days postoperatively. Severe AL was defined as Clavien–Dindo grade III or higher. The specific site of leakage was not uniformly recorded in the original medical charts, and therefore could not be consistently identified. Postoperative complications were also evaluated and classified using the Clavien–Dindo grading system. These variables were used for between-group comparisons and for identifying factors associated with AL in logistic regression analyses.

### ICG fluorescence imaging protocol

Intraoperative ICG fluorescence imaging was performed extracorporeally using near-infrared light excitation after the intravenous administration of 12.5 mg of ICG. Infrared light was directed at the targeted colon to assess blood perfusion. If poor or absent perfusion was identified, the transection line was adjusted to an area with adequate fluorescence. The anastomosis was performed at the initially planned transection line only when ICG imaging confirmed satisfactory perfusion. No ICG evaluation was performed on the anal side of the rectal stump. The intraoperative judgment of anastomotic perfusion quality using ICG fluorescence imaging was made by the attending surgeon on the basis of visual assessment. Although detailed criteria were not uniformly documented, only patients whose anastomoses were assessed as having good perfusion were included in this study.

### TA management

A silicon drainage tube (Create Medic Co., Yokohama, Japan) was inserted transanally and sutured to the anal verge. Specifically, the drainage tube had a 24-Fr drainage segment with three available effective lengths (80, 120, and 170 mm), selected by the operating surgeon on the basis of each patient’s anatomy. The decision to place a transanal drain and determine the position of the drainage tip was left to the discretion of the operating surgeon. The drain was generally left in place for five postoperative days. An open gravity drainage system was used in all cases.

A 24-Fr silicone drainage tube (Create Medic Co., Yokohama, Japan) was inserted transanally and sutured to the anal verge. The tube had three available effective lengths (80, 120, and 170 mm), and the selection, as well as the insertion depth, was determined by the operating surgeon according to patient anatomy and tumor location. The drainage tip was typically placed approximately 5–7 cm from the anal verge, in proximity to the anastomotic site.

### Statistical analysis

Continuous data are expressed as the mean ± standard deviation and median with interquartile range for normally and non-normally distributed variables, respectively. Categorical variables were compared using chi-squared or Fisher’s exact test. Univariate and multivariate logistic regression analyses were performed to identify AL-associated factors. The results are expressed as odds ratios (ORs) with 95% confidence intervals (CIs). Variables with a *p*-value < 0.05 in the univariate logistic regression analysis were included in the multivariate model. Statistical significance was set at *p* < 0.05. All analyses were performed using JMP Pro software (version 17.0, SAS Institute, Cary, NC, USA).

## Results

The baseline characteristics of patients in the TA and non-TA groups are summarized in Table 1. Several statistically significant differences were observed between the two groups. The proportion of patients with American Society of Anesthesiologists (ASA) physical status class III or IV was significantly higher in the TA group (19% versus 8%, *p* = 0.02). The use of minimally invasive surgery, including laparoscopic and robotic approaches, was more frequent in the TA group (91% versus 76%, *p* = 0.03). Preoperative therapy was more commonly performed in the non-TA group (0% versus 9%, *p* = 0.01).

**Table 1:** Baseline characteristics of patients in the TA and non-TA groups

	TA	Non-TA	*p*-Value
*n*	56	155	
Sex			0.94
Male, *n* (%)	31 (55)	85 (54)	
Female, *n* (%)	25 (45)	70 (73)	
Tumor location			
Upper, *n* (%)	10 (17)	38 (25)	
Middle, *n* (%)	22 (39)	62 (40)	
Lower, *n* (%)	24 (43)	55 (35)	
BMI, median (range), kg/m^2^	22.8 (15.4–37.8)	23.0 (19.1–48.7)	0.94
ASA class			0.02
I and II, *n* (%)	45 (80)	139 (91)	
III and IV, *n* (%)	11 (19)	13 (8)	
Age, median (range), years	69 (35–91)	67 (35–86)	0.76
cTstage			0.43
1 and 2, *n* (%)	27 (48)	62 (40)	
3 and 4, *n* (%)	29 (51)	93 (60)	
cNstage			0.48
N positive, *n* (%)	19 (33)	60 (38)	
N negative, *n* (%)	37 (66)	95 (61)	
Distant metastasis			0.48
Yes, *n* (%)	4 (7)	16 (10)	
No, *n* (%)	52 (92)	139 (90)	
Preoperative therapy			0.01
Yes, *n* (%)	0 (0)	15 (9)	
No, *n* (%)	56 (100)	139 (91)	
Procedure			0.22
HAR, *n* (%)	18 (32)	64 (41)	
LAR, *n* (%)	38 (68)	91 (58)	
LLND			0.85
Yes, *n* (%)	2 (3)	5 (3)	
No, *n* (%)	50 (96)	146 (97)	
Surgical approach			
Open	5 (8)	36 (23)	0.03
MIS			
Laparoscopy, *n* (%)	9 (16)	80 (52)	
Robotic, *n* (%)	42 (75)	39 (25)	
LCA preserved			0.001
Yes, *n* (%)	0 (0)	24 (15)	
No, *n* (%)	56 (100)	131 (85)	
Extended resection			1
Yes, *n* (%)	4 (7)	11 (7)	
No, *n* (%)	52 (93)	144 (93)	
Concomitant surgery			0.76
Yes, *n* (%)	4 (7)	13 (8)	
No, *n* (%)	52 (93)	142 (92)	
Operative time, median (range), min	225 (143–466)	223 (130–522)	0.35
Blood loss, median (range), mL	5 (0–1291)	10 (0–1318)	0.07
Transfusion			0.56
Yes, *n* (%)	0 (0)	3 (2)	
No, *n* (%)	56 (100)	152 (98)	
Conversion			0.68
Yes, *n* (%)	1 (2)	4 (3)	
No, *n* (%)	49 (98)	125 (97)	
AV, median (range), cm	7 (3–15)	7 (3–20)	0.97
Bulky tumor			0.43
Yes, *n* (%)	1 (2)	1 (1)	
No, *n* (%)	49 (98)	149 (99)	

Some variables have missing data; percentages are based on available data

*TA* transanal tube, *ASA* American Society of Anesthesiologists, *LAR* low anterior resection, *HAR* high anterior resection, *LLND* lateral lymph node dissection, *MIS* minimally invasive surgery, *BMI* body mass index, *LCA* left colic artery, *AV* distance from the anal verge

The postoperative outcomes are summarized in Table 2. The overall complication rate was significantly lower in the TA group compared with the non-TA group (28% versus 41%, *p* = 0.04). Severe complications (Clavien–Dindo grade III or higher) were also significantly less frequent in the TA group (5% versus 19%, *p* = 0.01).

**Table 2:** Surgical results

	TA	Non-TA	*p*-Value
*n*	56	155	
Postoperative complication, *n* (%)			
Overall	16 (28)	65 (41)	0.07
Severe	3 (5)	30 (19)	0.01
Anastomotic leakage			
Overall	3 (5)	28 (18)	0.02
Severe	1 (1)	15 (10)	0.04
Day of AL occurrence, median day (range)	3(2–6)	3(0–8)	0.72
Postoperative ileus	0 (0)	10 (11)	1
Wound infection	1 (1)	12 (8)	0.77
Abscess	0 (0)	3 (2)	1
Reoperation within 30 days	2 (3)	9 (5)	0.51
Hospital stay, days	7 (6–76)	9 (5–67)	0.003

Severe complications were defined as Clavien–Dindo classification grade III or higher

The incidence of overall AL was significantly lower in the TA group (5% versus 18%, *p* = 0.008), and the rate of severe AL was also reduced (1% versus 10%, *p* = 0.02). There was no statistically significant difference in the reoperation rate between the two groups (3% versus 5%).

Univariate logistic regression analysis was performed to identify risk factors associated with AL. Among the tested variables, TA placement was significantly associated with a reduced risk of AL (OR 0.25; 95% CI 0.07–0.88; *p* = 0.02). Male sex (OR 4.07; 95% CI 1.59–10.40; *p* = 0.001) and the presence of lateral lymph node dissection (LLND) (OR 4.90; 95% CI 1.03–23.16; *p* = 0.02) were significantly associated with an increased risk of AL. Subsequently, these three variables were included in the multivariate logistic regression model. In the multivariate analysis, TA placement remained an independent protective factor against AL (OR 0.27; 95% CI 0.06–0.86; *p* = 0.02), while male sex was identified as an independent risk factor (OR 4.7; 95% CI 1.82–14.63; *p* = 0.001). LLND showed a trend toward increased risk but did not reach statistical significance after adjustment (OR 4.90; 95% CI 1.03–23.16; *p* = 0.07) (Table 3).

**Table 3:** Risk factor of anastomotic leakage

Variables	Univariate		Multivariate	
	OR (95% CI)	*p*-Value	OR (95% CI)	*p*-Value
TA	0.25 (0.07–0.88)	0.02	0.27 (0.06–0.86)	0.02
Sex, male	4.07 (1.59–10.40)	0.001	4.7 (1.82–14.63)	0.0009
BMI ≥ 30 kg/m^2^	0	0.3		
ASA ≥ III	0.79 (0.22–2.84)	0.72		
Age ≥ 75 years	0.47 (0.15–1.42)	0.24		
Stage III–IV	1.04 (0.53–2.04)	0.9		
Preoperative therapy	2.54 (0.32–20.08)	0.7		
LLND	4.90 (1.03–23.16)	0.02	4.64 (0.78–26.57)	0.08
MIS	0.48 (0.20–1.16)	0.13		
LCA preserved	0.80 (0.22–2.86)	0.73		
Extended resection	0.88 (0.18–4.10)	0.86		
Operative time ≥ 240 min	2.06 (0.94–4.51)	0.06		
Blood loss ≥ 1000 mL	4.70 (0.99–22.22)	0.06		
AV < 5 cm	0.52 (0.11–2.43)	0.4		
Tumor size ≥ 5 cm	5.82 (0.35–95.80)	0.27		

*TA* transanal tube, *ASA* American Society of Anesthesiologists, *LLND* lateral lymph node dissection, *MIS* minimally invasive surgery, *BMI* body mass index, *LCA* left colic artery, *AV* distance from the anal verge, *OR* odds ratio, *CI* confidence interval

A subgroup analysis was performed to evaluate the effect of TA placement based on tumor location (upper, middle, or lower rectum). The incidence of AL was significantly lower in the TA group compared with the non-TA group among patients with middle rectal cancer (0% versus 19%, *p* = 0.03). No statistically significant differences were observed in the upper or lower rectal cancer subgroups (Table 4).

**Table 4:** Effect of TA placement on anastomotic leakage stratified by tumor location

Tumor location	Group	Anastomotic leakage (%)	*p*-Value
Upper rectum	TA	0 (0)	
	Non-TA	6 (15)	0.32
Middle rectum	TA	0 (0)	
	Non-TA	12 (19)	0.03
Lower rectum	TA	3 (12)	
	Non-TA	10 (18)	0.74

*TA* transanal tube

## Discussion

Although TA placement has been suggested as a method to mitigate the risk of AL, its clinical efficacy remains controversial. One major confounding factor in previous studies is the inconsistency in assessing anastomotic perfusion.

In the present study, we evaluated the effect of TA placement on AL under standardized perfusion conditions, using intraoperative ICG fluorescence imaging to confirm adequate anastomotic blood flow. Our results demonstrated that TA placement was associated with a significantly lower incidence of AL, particularly in patients with middle rectal cancer. This suggests that when sufficient perfusion is ensured, TA placement may play a crucial role in preventing AL after rectal cancer surgery.

Several studies have investigated the effectiveness of TA placement in mitigating the risk of AL following rectal cancer surgery. Kuk et al. [11] evaluated the data of 556 patients with rectal cancer who underwent low anterior resection using the double stapling technique and found that the TA group had a significantly lower AL rate (1.9%) than the control group (5.6%) (*p* = 0.03). Wang et al. [12] reported a beneficial effect of TA use in reducing AL post-low anterior resection. In their clinical study of 220 patients with rectal cancer who underwent laparoscopic low anterior resection using the double stapling technique, a significant reduction in AL rates from 6.0% in the control group to 3.3% in the TA group was observed (*p* < 0.05). These results may further support the evidence regarding the potential benefit of TA in reducing AL risk in rectal cancer surgery, highlighting its role in maintaining anastomotic integrity by providing continuous decompression. However, a study by Tamura et al. [13] involving 157 patients who underwent elective low anterior resection showed no significant difference in symptomatic AL between the TA and non-TA groups (7.6% versus 10.3%, *p* = 0.559). Although a larger sample size may demonstrate a benefit, the study concluded that no significant effect was observed. A large-scale multicenter trial in China [14], including 560 patients undergoing laparoscopic low anterior resection for mid–low rectal cancer without preoperative radiotherapy also showed no significant difference in AL rates or grades between the TA and non-TA groups (6.4% versus 6.8%, *p* = 0.87; grade B, 5.0% versus 3.9%, and grade C, 1.4% versus 2.9%, *p* = 0.43). The trial concluded that TA placement may not provide any benefit in preventing AL. However, these studies did not describe specific methods for intraoperative confirmation of anastomotic perfusion, such as ICG fluorescence imaging. Therefore, it remains unclear whether adequate blood flow was ensured prior to anastomosis, which may partly explain the inconsistency in the reported effectiveness of TA placement.

In our study, we utilized ICG fluorescence imaging to confirm sufficient anastomotic perfusion before completing the anastomosis. This technique has been increasingly adopted in colorectal surgery to prevent ischemic complications, such as anastomotic leakage [5]. Without ensuring adequate perfusion, the benefit of TA placement alone might be insufficient to prevent AL, as compromised vascularity remains a dominant risk factor. Moreover, in the present study, TA placement was evaluated under conditions where adequate perfusion was confirmed, highlighting its potential role when ischemic factors are minimized. Importantly, all included patients had good anastomotic perfusion confirmed intraoperatively by ICG fluorescence imaging. The fact that AL still occurred despite this standardized confirmation suggests that providing adequate blood flow alone may not be sufficient to prevent AL, further underscoring the potential value of TA placement under optimized perfusion conditions.

The identification of male sex as a risk factor aligns with previous findings [15] and may be attributed to anatomical challenges. Specifically, male patients tend to have narrower and deeper pelvic cavities, which can complicate pelvic dissection and limit maneuverability during rectal transection and anastomosis. These technical difficulties may increase the risk of tension, misalignment, or suboptimal perfusion at the anastomotic site, contributing to a higher incidence of AL. Notably, TA placement appears to function not as an intraoperative intervention but as a postoperative management approach that modifies the local environment. By providing continuous decompression at the anastomotic site, TA placement may help maintain low intraluminal pressure during the critical early healing period. This hypothesis is consistent with the observed protective effect of TA placement and supports its role in optimizing the postoperative environment to prevent anastomotic disruption.

Tamura et al. [16] conducted a meta-analysis to assess the effectiveness and safety of TA placement, reporting mixed outcomes. While observational studies demonstrated a significant reduction in AL rates (OR 0.45, 95% CI 0.31–0.64), randomized controlled trials showed no clear benefit. This discrepancy suggests the need for further investigation to identify patient populations that may particularly benefit from TA placement and to optimize the conditions under which it is most effective.

In the present study, we performed a subgroup analysis on the basis of tumor location to assess the effectiveness of combining ICG fluorescence imaging with TA placement in preventing AL. The results indicated that the combination was particularly beneficial in patients with middle rectal cancer. In contrast, lower rectal cancer cases showed limited benefit, which may be partly attributed to technical limitations—specifically, in approximately 25% of these cases, the tip of the transanal drainage tube was located proximal to the anastomosis. Although this information was not presented in the main tables due to missing data in some cases, it was available for a subset of patients and may offer a plausible explanation for the reduced efficacy observed in lower rectal tumors.

This study has some limitations. First, differences in baseline characteristics between the ICG + TA placement and control groups may reflect shifts in referral patterns or patient selection criteria over time. Second, subgroup analyses, particularly those based on tumor location, were limited by small sample sizes, especially in lower rectal cancer cases, making it difficult to draw definitive conclusions. Third, the number of patients in the TA group was insufficient precluding the use of propensity score matching or multivariate adjustment. This may have led to unmeasured confounding when comparing outcomes between groups. Fourth, although the TA placement protocol was standardized in terms of device and general practice, detailed documentation on drain duration and management (e.g., early removal due to complications) was not consistently available due to the retrospective nature of the study. This may have introduced variability in the actual exposure to drainage among patients. Despite these limitations, the observed trends are biologically plausible and consistent with the proposed mechanisms. Therefore, future multicenter, prospective randomized controlled trials with standardized intervention protocols and adjustments for temporal factors are required to validate and build upon our findings.

In conclusion, our findings suggest that the combination of ICG fluorescence angiography and TA placement, aimed at ensuring adequate anastomotic perfusion and targeted transanal decompression, may be associated with a reduced risk of AL following rectal cancer surgery.

## Data Availability

No datasets were generated or analyzed during the current study.
